# The first two blind troglobitic spiders of the genus *Ochyrocera* from caves in Floresta Nacional de Carajás, state of Pará, Brazil (Araneae, Ochyroceratidae)

**DOI:** 10.3897/zookeys.1031.62181

**Published:** 2021-04-15

**Authors:** Antonio D. Brescovit, Robson de A. Zampaulo, Igor Cizauskas

**Affiliations:** 1 Laboratório de Coleções Zoológicas, Instituto Butantan, Av. Vital Brasil, 1500, Butantã, São Paulo, 05503-900, Brazil Instituto Butantan São Paulo Brazil; 2 Gerencia de Licenciamento Ambiental e Espeleologia, Vale SA, Avenida Doutor Marco Paulo Simon Jardim, 34006-200 Nova Lima, Minas Gerais, Brazil Gerencia de Licenciamento Ambiental e Espeleologia Nova Lima Brazil; 3 Organização de Apoio à Pesquisa da Biodiversidade (OAPBio), Rua Frei Inácio da 238, 05362-040, São Paulo, Brazil Organização de Apoio à Pesquisa da Biodiversidade São Paulo Brazil

**Keywords:** Amazonian region, blind ground weaver spider, Synspermiata, taxonomy, troglobite

## Abstract

The first two anophthalmic species of spiders of the genus *Ochyrocera* Simon, 1892, are described for caves located in the iron formation of Floresta Nacional (FLONA) de Carajás in southeastern Pará State, Brazil. The caves are located in the municipalities of Parauapebas and Canaã dos Carajás, in the eastern portion of the Amazon Forest domain. *Ochyrocera ritxoco***sp. nov.** and *O. ritxoo***sp. nov.** are described based on males and females. The species have similar body characteristics with the total absence of eyes and complete depigmentation, characteristics that indicate possible evolution in subterranean environments , and thus are classified as troglobites. Each species is associated with a single geomorphological unit (mountain range), with *Ochyrocera ritxoco***sp. nov.** being restricted to caves of Serra Norte (North Mountain) and *O. ritxoo***sp. nov.** to caves of Serra Sul (South Mountain). Both species were collected in aphotic zones of the caves. Small and tangled webs of *O. ritxoco***sp. nov.** were observed under blocks of stone in the soil or in cracks of the walls.

## Introduction

Ochyroceratidae currently contains 10 genera and 166 species (World Spider Catalog 2020), with the recent elevation of Psilodercidae reducing its diversity by half (Wunderlich 2008). Despite this, the distribution the family can be considered wide, occurring in tropical areas of the Neotropical, African and Indo-Pacific regions. Its species are common on the ground and in cave environments, with sizes not greater than 2 mm, and having six eyes and long, thin legs (Jocqué and Dippenaar-Schoeman 2006).

Although many ochyroceratids live in hypogean environments, few have morphological specializations related to life in caves, which would characterize them as troglomorphic. We highlight here, at least five species of the family whose members possess some type of troglomorphism, namely: *Speocera caeca* described by Deeleman-Reinhold (1995) from Indonesia, *Speocera eleonorae* Baptista, 2003 from Brazil, *Theotima pura* Gertsch, 1973 and *Theotima martha* Gertsch, 1977, which occur in caves on the Yucatan Peninsula, Mexico, and *Ochyrocera peruana* Ribera, 1978 from Peru. Only two species are blind spiders (*S. caeca* and *T. pura*), while the others are characterized by reduced or small size of the eyes, depigmented body and long thin legs (Gertsch 1977).

In this work, we describe two new troglobitic species of the ochyroceratid genus *Ochyrocera*. These species represent the first blind and depigmented members of the genus, which currently possesses 50 species worldwide (World Spider Catalog 2020).

The two species were collected from iron formation caves in FLONA de Carajás (Carajás National Forest), state of Pará, northern Brazil and expand the diversity of spiders known for this ferruginous region (Brescovit et al. 2018).

## Materials and methods

### Taxonomic descriptions

Specimens are deposited in the following collections (abbreviation and curator in parentheses): Instituto Butantan, São Paulo (IBSP, A.D. Brescovit) and Museu Paraense Emílio Goeldi, Belém (MPEG, A.B. Bonaldo).

Morphological terms follow Brescovit et al. (2018), except for macrosetae of endites which follow Baert (2014). Descriptions and measurements were performed using a Leica 165C stereomicroscope, while photographs were taken with a Leica DFC 500 digital camera mounted on a Leica MZ16A stereomicroscope. Focal range images were made using Leica Application Suite software, version 2.5.0. Total and femur lengths were measured in lateral view without detaching any part from the specimen. All measurements are in millimeters. Female genitalia were excised with a sharp needle and photographed mounted on Hoyer´s microscope slides. For scanning electron microscopy (SEM), body parts were dehydrated in a series of graded ethanol washes (80% to 100%), critical point dried, mounted on metal stubs using adhesive copper tape and nail polish for fixation and covered with gold. SEM images were taken with FEI Quanta 250 and LEO 1450VP scanning electron microscopes, at Laboratório de Biologia Celular of Instituto Butantan, São Paulo and Museu Paraense Emílio Goeldi, Belém, respectively.

### Study area

The caves where the spiders were sampled are inserted in iron formations located in the Carajás area in southeast state of Pará, in the eastern region of the Amazon Forest in Brazil (Fig. 10). The caves are within the FLONA de Carajás (Brazilian System of Conservation Units), which encompasses approximately 411 thousand hectares and includes parts of the municipalities of Parauapebas, Canaã dos Carajás and Água Azul do Norte. In the region of the park there is a mosaic of protected areas forming a continuous area of 1.31 million hectares of preserved forest (Rolim et al. 2006), which is surrounded by pastures that replaced original forest (Campos and Castilho 2012; Martins et al. 2012; Carmo and Jacobi 2013). The park area mainly comprises forest formations (ombrophilous or seasonal) and only 5% of campo rupestre (rocky/rupestrian fields), which develops on the laterite plates (crusts) of high areas of the region (Campos and Castilho 2012).

## Taxonomy

**Family Ochyroceratidae Fage, 1912**

### Genus *Ochyrocera* Simon, 1892

#### Ochyrocera ritxoco sp. nov.

Taxon classificationAnimaliaAraneaeOchyroceratidae

4FE7FB76-7986-5ABA-B756-06F44AE07506

http://zoobank.org/3A17CF00-CFE6-482A-89B7-2CF243171883

**Figure 1.**
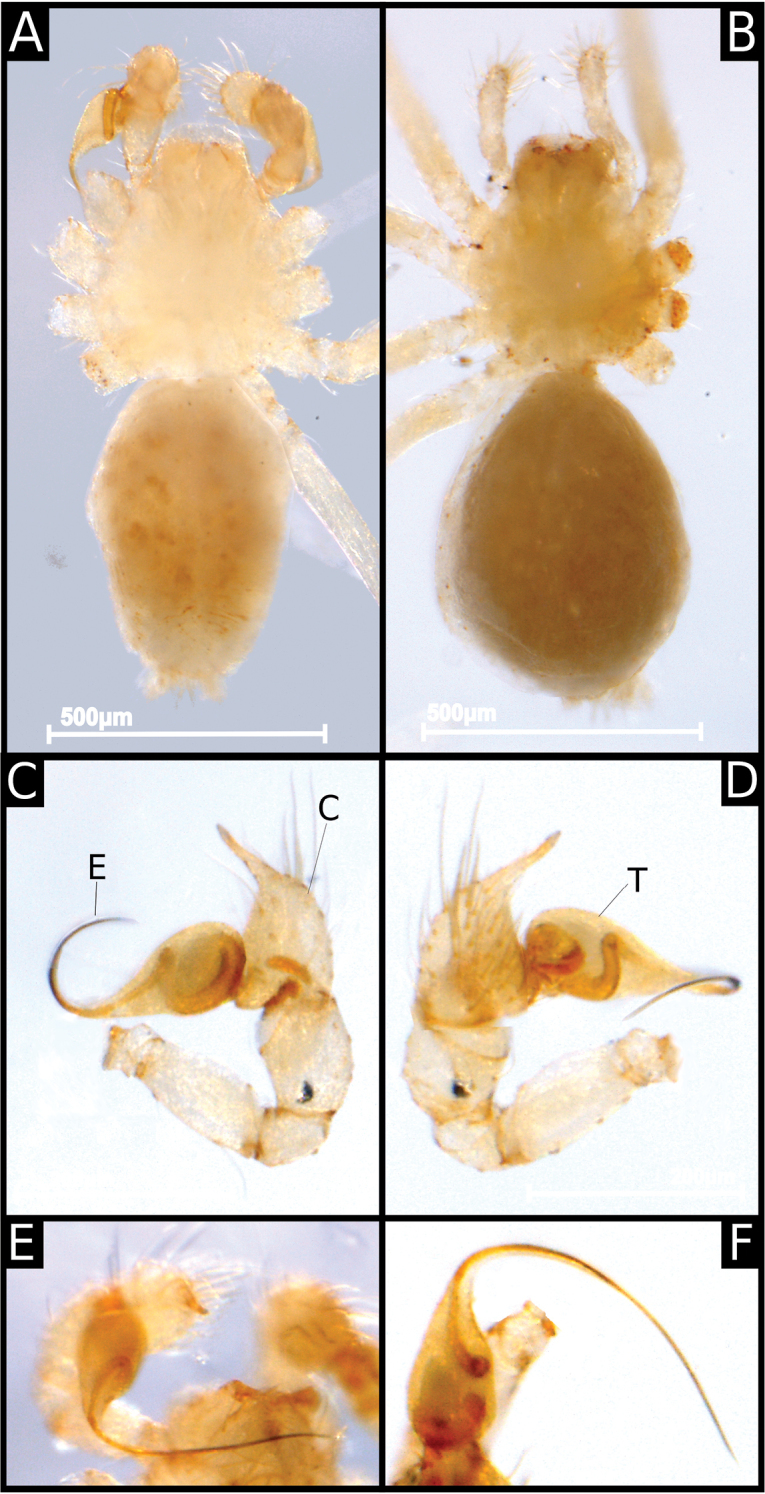
*Ochyrocera ritxoco* sp. nov., male IBSP 186160 (**A, C–F**), female IBSP 186146 (**B**) **A, B** habitus, dorsal view **C** left male palp, retrolateral view **D** same, prolateral view **E** right male palp, ventral view **F** same, dorsal view. Abbreviations: C = cymbium, CE = cymbial extension, E = embolus, T = tegulum.

**Figure 2.**
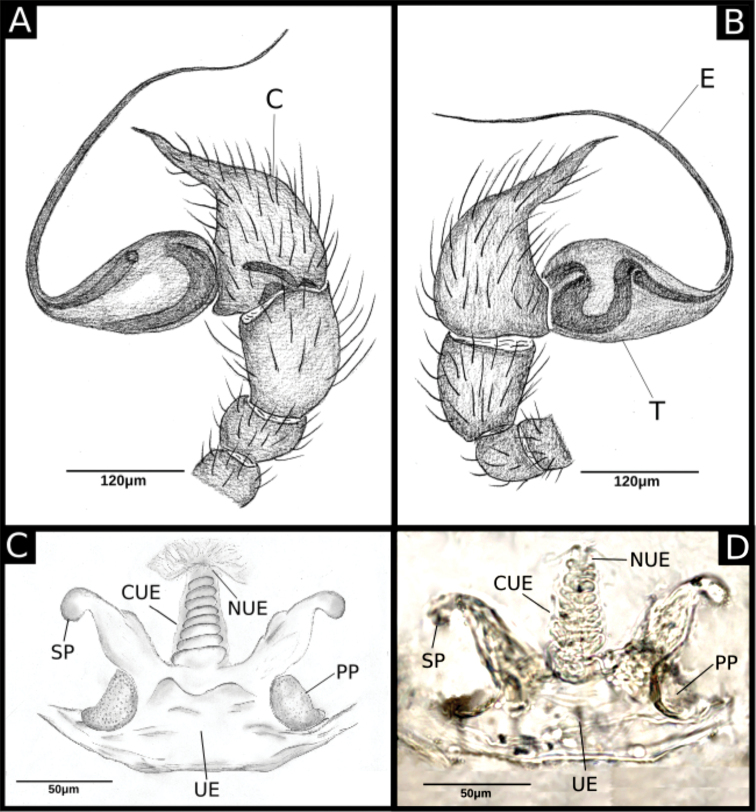
*Ochyrocera ritxoco* sp. nov. (**A, B**) **A** left male palp IBSP 186149, retrolateral view **B** same, prolateral view (**C, D) C** female genitalia IBSP 186149, enzyme cleared, dorsal view **D** same, dorsal view. Abbreviations: C = cymbium, CUE = columnar uterus externus, E = embolus, NUE = neck of uterus externus, PP = pore-plate, SP = spermathecae, T = tegulum, UE = uterus externus.

**Figure 3.**
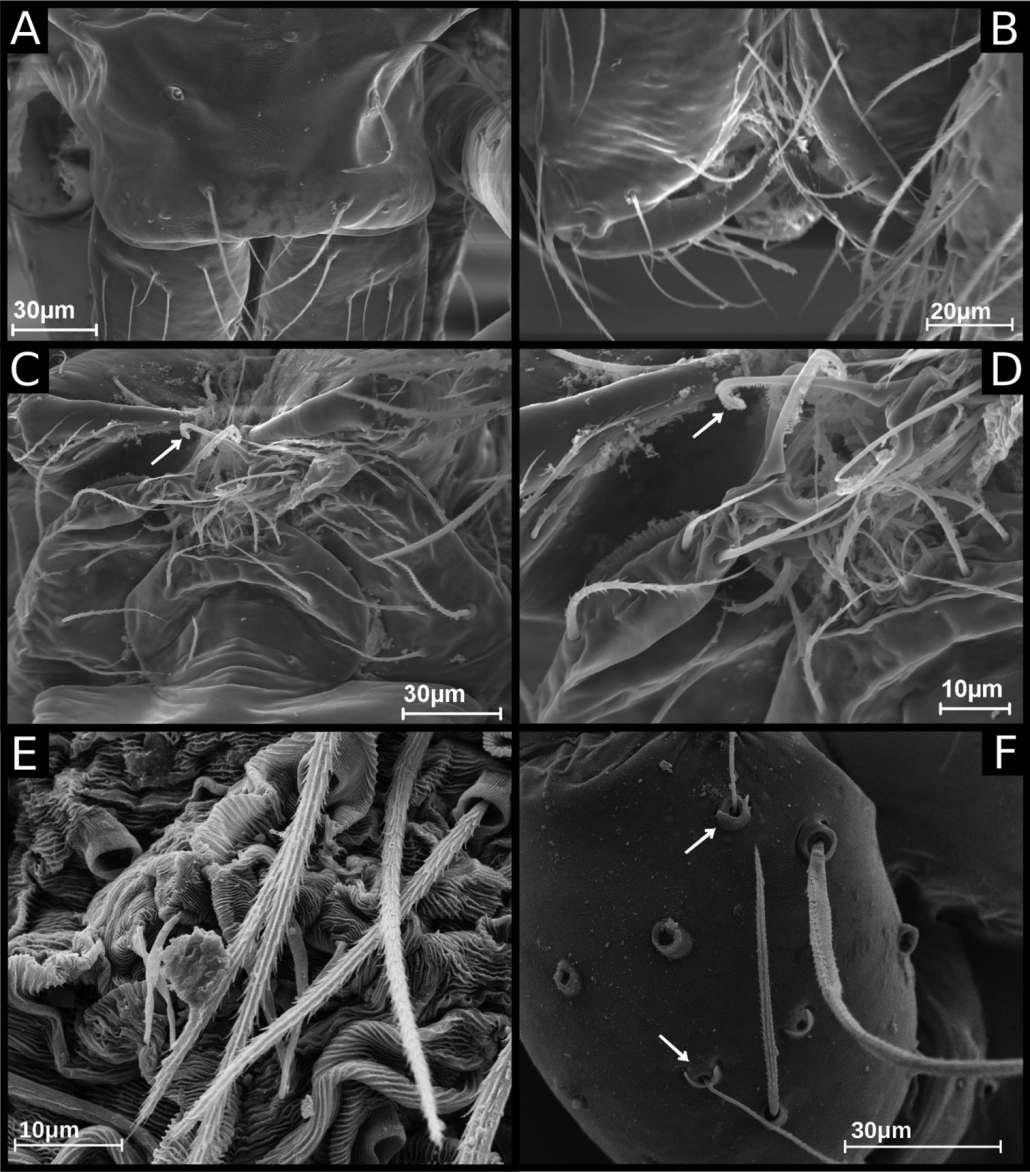
SEM images of *Ochyrocera ritxoco* sp. nov., male IBSP 260307 (**A–F**) **A** carapace, dorsal view **B** chelicerae, frontal view **C** endites and labium, ventral view **D** crosier-like macrosetae (arrow, detail) **E** epiandrous area, abdomen, ventral view **F** male palp, tibia (arrows, trichobothria), dorsal view.

**Figure 4.**
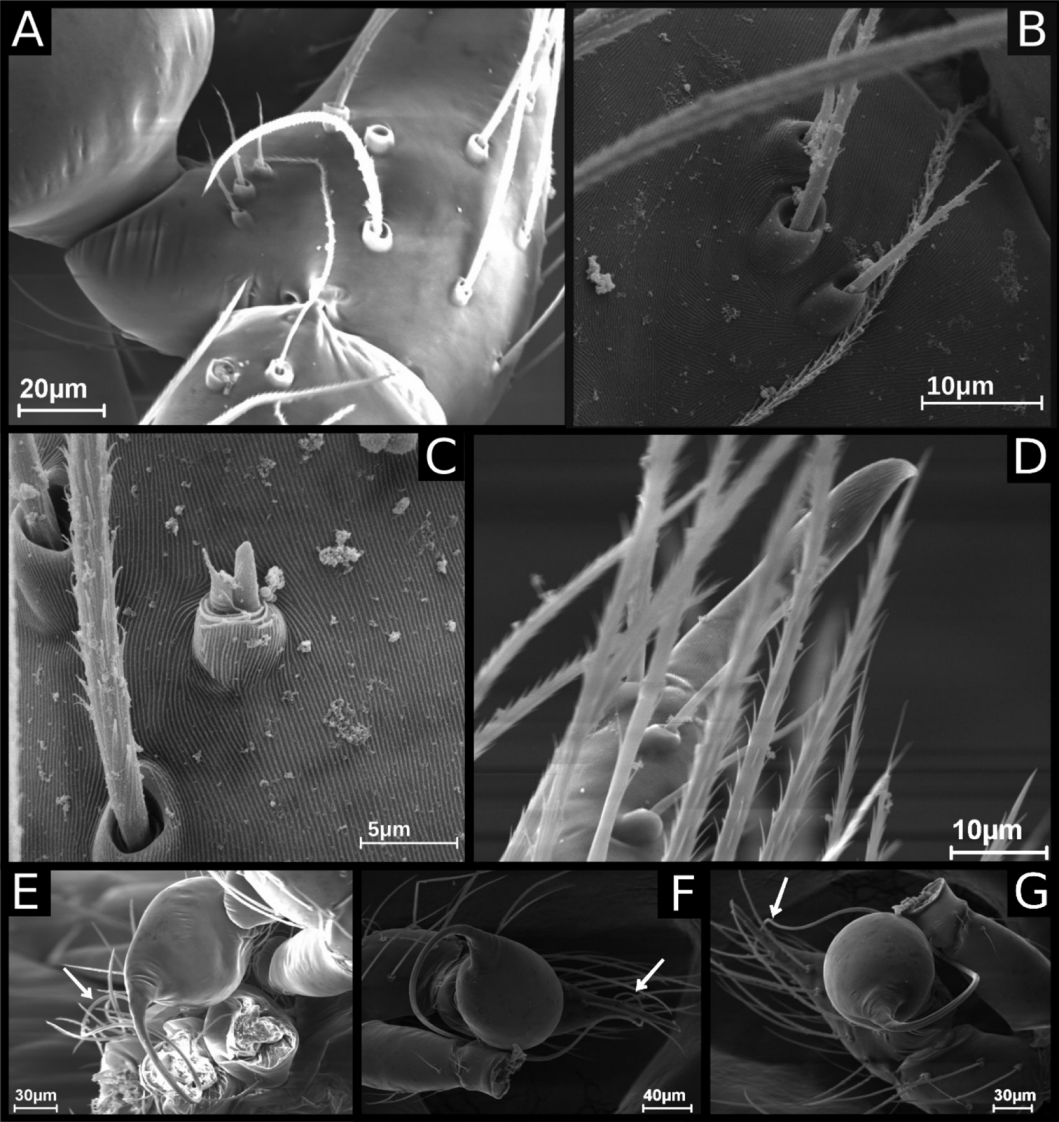
SEM images of *Ochyrocera ritxoco* sp. nov., male IBSP 260307 (**A–G**) **A** cymbium, retrolateral view **B** same, basal macrosetae, retrolateral view **C** same, tarsal organ, detail, retrolateral view **D** same, apex detail **E–G** male palp, arrows indicating the embolus **E** retrolateral view **F** same, prolateral view **G** same, frontal view.

**Figure 5.**
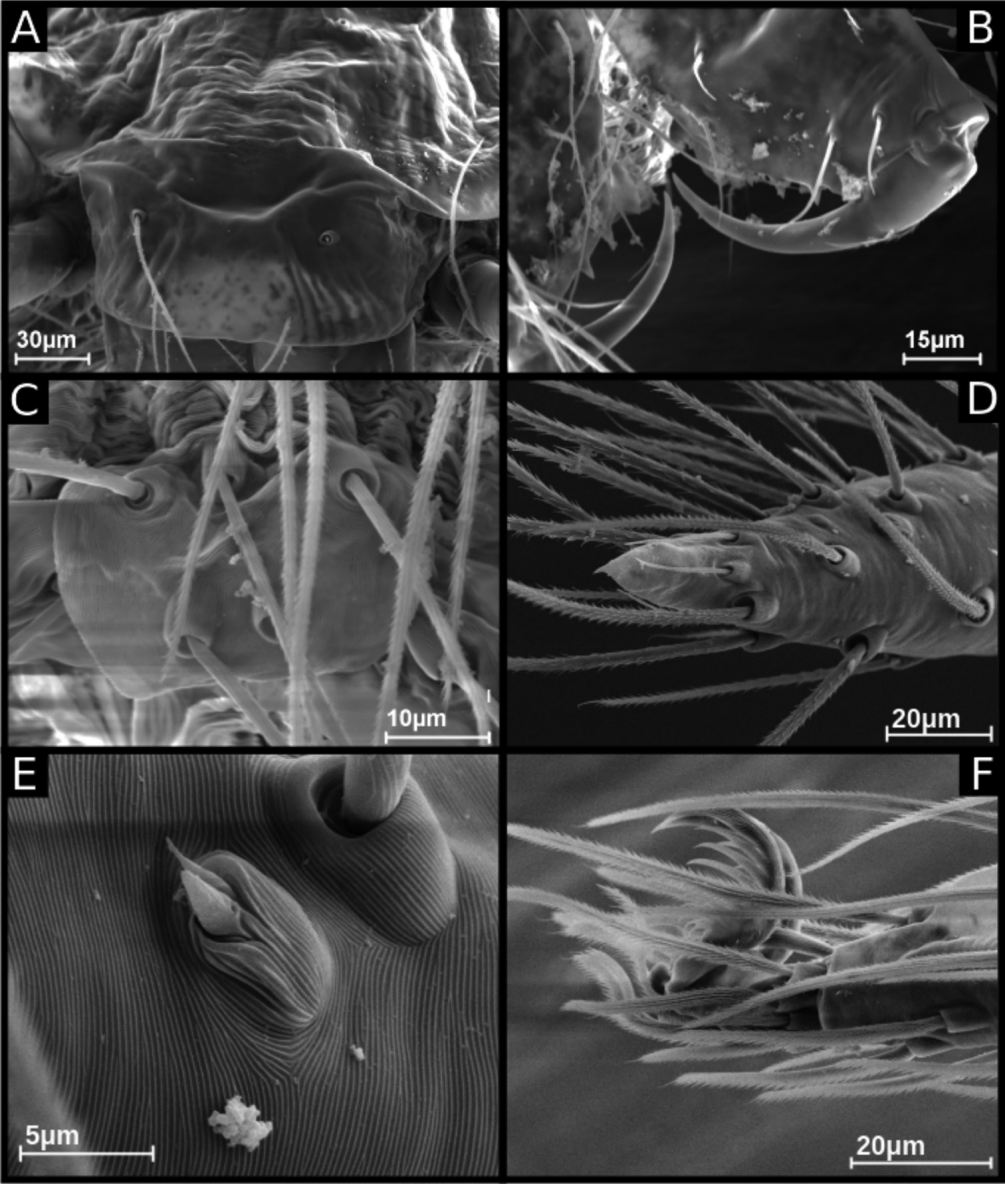
SEM images of *Ochyrocera ritxoco* sp. nov., female IBSP 260307 (**A–F**) **A** carapace, dorsal view **B** chelicerae, frontal view **C** colulus, ventral view **D** pedipalp, distal, prolateral view **E** same, tarsal organ **F** leg IV, claw, prolateral view.

**Figure 6.**
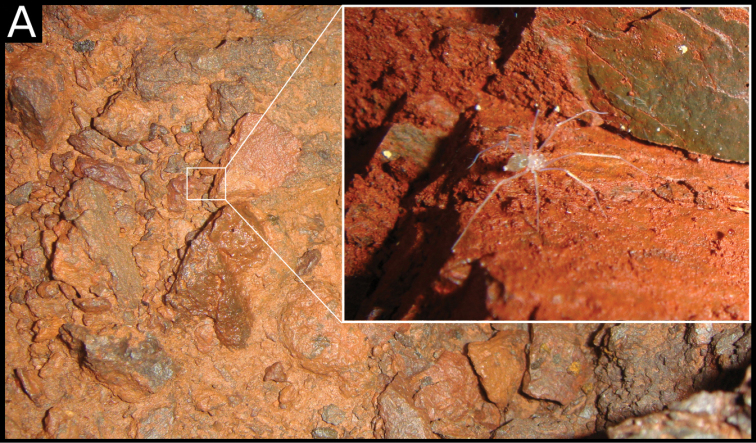
*Ochyrocera ritxoco* sp. nov., female in webs under rocks on the ground in the Cave N4WS_0067.

Figs 1 , 2 , 3 , 4 , 5 , 6 , 10

##### Type material.

***Holotype*** male from Cave N1_0103 (GEM-1301 or Cipó cave; 6°0'13"S, 50°17'55"W), FLONA de Carajás, Parauapebas, Pará, Brazil, 28/XI-03/X/2007, R. Andrade et al. col. (IBSP 115497). ***Paratype*** female from Cave N1_0075 (GEM_1273 or Piranha cave; 6°1'14"S, 50°16'49"W), FLONA de Carajás, Parauapebas, Pará, Brazil, 28/XI–03/X/2007, R. Andrade et al. col. (IBSP 115499).

##### Other material examined.

Brazil. Pará: Parauapebas, FLONA de Carajás, Cave N1_0015 (GEM-1211) (6°2'2"S, 50°16'16"W), 11/VI–02/VII/2014, 1♀ (IBSP 186123); 1♀ (IBSP 186124); 1♂ (IBSP 186125); 2♀ (IBSP 186126); Cave N1_0016 (GEM-1212) (6°1'10"S, 50°16'41"W), 04/IX–06/X/2014, 1♀ (IBSP 186130); 02–29/IV/2015, 2♀ (IBSP 186153); 3♀ (IBSP 186154); 1♀ (IBSP 186155); Cave N1_0055 (GEM-1253) (6°1'12"S, 50°16'43"W), 07–28/I/2015, 1♀ (MPEG 37086, ex IBSP 186147); Cave N1_0056 (GEM-1254) (6°1'11"S, 50°16'44"W), 07–28/I/2015, 1♂ (MPEG 37087, ex IBSP 186148); 1♂ 1♀ (IBSP 186149); Cave N1_0060 (GEM-1258) (6°1'12"S, 50°16'41"W), 11/VI–02/VII/2014, 1♀ (IBSP 186127); 1♀ (IBSP 186128); 07–28/I/2015, 1♀ (IBSP 186150); 1♀ (IBSP 186151); 1♂ 1♀ (MPEG 37088, ex IBSP 186152); Cave N1_0062 (GEM-1260) (6°1'10"S, 50°16'44"W), 04/IX–06/X/2014, 1♀ (IBSP 186131); 1♀ (IBSP 186132); 2♀ (IBSP 186133); 1♀ (IBSP 186134); 1♂ 2♀ (IBSP 186135); 02–29/IV/2015, 2♀ (IBSP 186157); 3♂ 1♀ (IBSP 186156); 1♀ (IBSP 186158); Cave N1_0073 (GEM-1271) (6°1'13"S, 50°17'17"W), 02–29/IV/2015, 1♀ (IBSP 186159); 1♂ (IBSP 186160); 1♀ (IBSP 186161); 1♀ (IBSP 186162); Cave N1_0084 (GEM-1282) (6°1'7"S, 50°17'1"W), 11/VI–02/VII/2014, 1♀ (IBSP 186129); Cave N1_0101 (GEM-1299) (6°1'9"S, 50°16'46"W), 04/IX–06/X/2014, 1♂ (IBSP 186136); 1♀ (IBSP 186137); 1♀ (IBSP 186138); 1♂ (IBSP 186139); 2♀ (IBSP 186140); 1♂ (IBSP 186141); 1♂ (IBSP 186142); Cave N1_0240 (6°1'19"S, 50°16'26"W), 04/IX–06/X/2014, 1♂ (IBSP 186143); 1♀ (IBSP 186144); 1♂ (IBSP 186145); 1♀ (IBSP 186146); 02–29/IV/2015, 1♀ (IBSP 186163); 1♂ 1♀ (IBSP 186164); all collected by Equipe Carste; Cave N4WS_0067 (GEM-1846) (6°04'22"S, 50°11'30"W), 18/XI–01/XII/2010, F.P. Franco & C.A.R. Souza et al. col., 1♂ (IBSP 174069); 2♀ (IBSP 174070); Piranha, Cave N1-75 (6°1’14"S, 50°16’49"W), 28/IX–03/X/2007, 1♂ 2♀ (IBSP 260307; 1♂ SEM; 1♀ SEM, ex IBSP 115499); all collected by R. Andrade et al.

##### Diagnosis.

**Figure 7.**
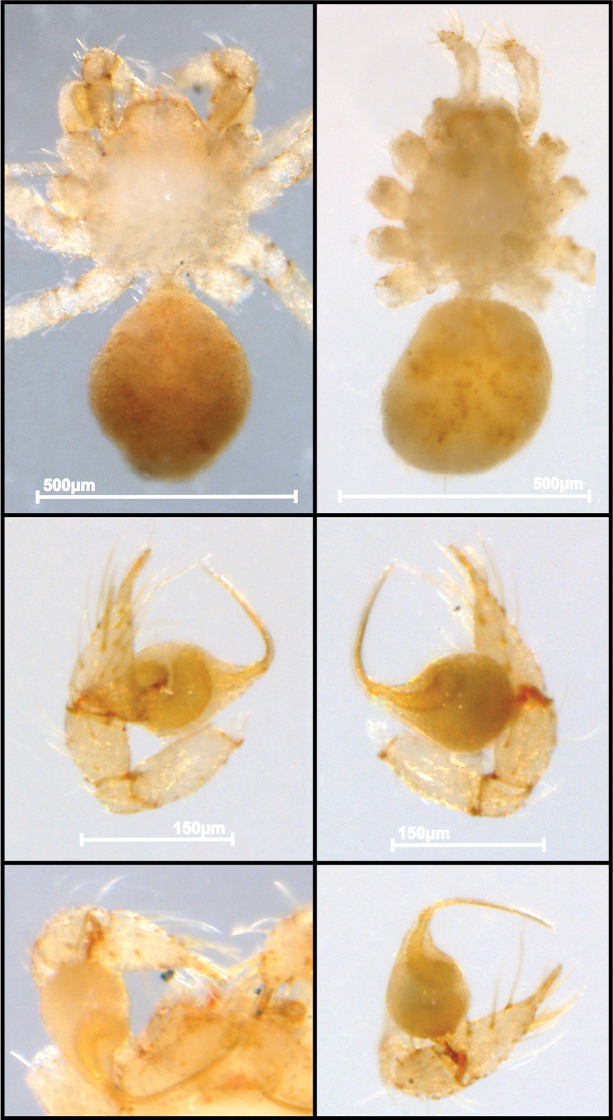
*Ochyrocera ritxoo* sp. nov., male IBSP 193194 (**A, C–F**), female IBSP 193196 (**B**) **A, B** habitus, dorsal view **C** left male palp, retrolateral view **D** same, prolateral view **E** right male palp, dorsal view **F** same, retro-ventral view. Abbreviations: C = cymbium, CE = cymbial extension, E = embolus, T = tegulum.

**Figure 8.**
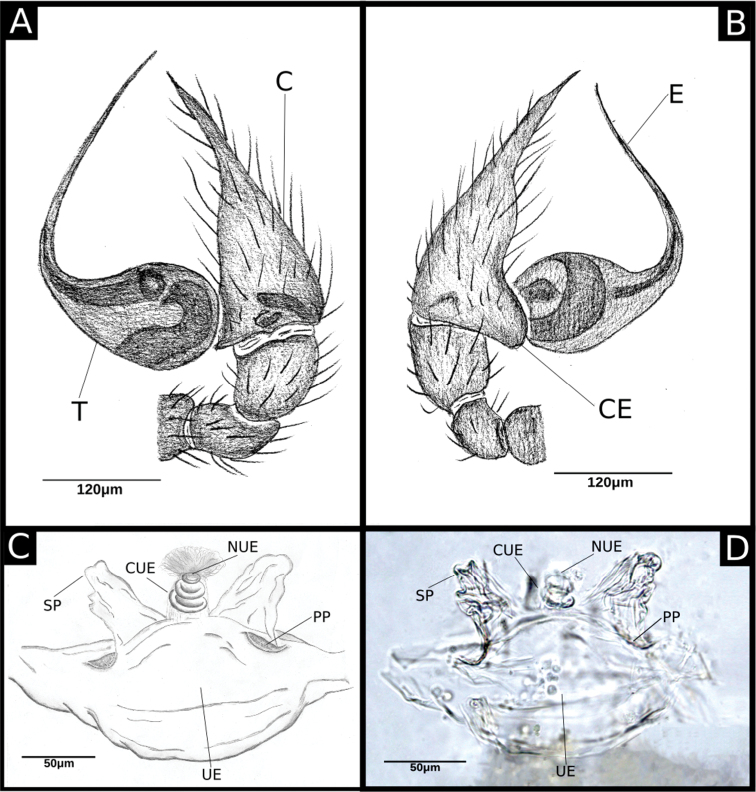
*Ochyrocera ritxoo* sp. nov. (**A, B**) **A** left male palp IBSP 193194, retrolateral view **B** same, prolateral view (**C, D) C** female genitalia IBSP 1741071, enzyme cleared, dorsal view **D** same, dorsal view. Abbreviations: C = cymbium, CE = cymbial extension, CUE = columnar uterus externus, E = embolus, NUE = neck of uterus externus, PP = pore-plate, SP = spermathecae, T = tegulum, UE = uterus externus.

*Ochyrocera ritxoco* sp. nov. is distinguished from *O. ritxoo* sp. nov. by having an elongated embolus, which is two times longer than the bulb in the male palp (Figs 1F, 2A, B, 4E–G), while the embolus is one times longer than the bulb in *O. ritxoo* sp. nov. (Fig. 7C, D); a long and globose distal area in the spermathecae and an elongated columnar uterus externus with approximately eight internal chambers in the female genitalia (Fig. 2C, D); while *O. ritxoo* sp. nov. have a triangular distal area in the spermathecae and a shorter columnar uterus externus with 3–4 internal chambers in the female genitalia (Fig. 8C, D).

##### Description.

**Male** (Holotype). Total length 1.1. Carapace length 0.45; ovoid, narrowing gradually anteriorly, yellowish and bright, pars cephalic flat, fovea absent (Figs 1A, 3A). Clypeus with two pairs of long bristles (Fig. 3A). Eyes absent. Chelicerae light yellow with orange fang, promargin with three teeth attached to a very long lamina (Fig. 3B), retromargin without teeth. Endites light yellow with large serrula with more than 30 denticles, distal macrosetae paired and crosier-like, many multifid macrosetae present (Fig. 3C, D). Labium cream-colored, rounded with 8–10 setae with an enlarged basally (Fig. 3C). Sternum light yellow. Legs cream-colored, formula 1423, total length I 4.5, II 3.8, III 3.1, IV 4.0. Male palp with palpal femur length 0.04, palpal tibia almost as long as wide with two long dorsal trichobothria (Fig. 3F), cymbium enlarged basally, narrowed distally, bearing elongated cuspule (Fig. 4D), three setae on projected bases retrolaterally (Fig. 4A, B), elongated tarsal organ subdistally (Fig. 4C), no basal setae on the rounded cymbial prolateral extension, bulb oval, embolus filiform, sinuous and at least twice as long as the cymbium (Figs 1F, 2A, B, 4E–G). Abdomen length 0.50, oval, uniformly gray. Six epiandrous spigots with a short base (Fig. 3E)

**Female** (Paratype IBSP 115499). Total length 1.15. Carapace length 0.55 as in male with light yellowish pattern (Figs 1B, 5A). Pedipalp without claw, with conical tip and subdistal trichobothrium (Fig. 5D–E). Clypeus, eyes, chelicerae (Fig. 5B), sternum, endites, and labium as in male. Legs as in male, formula 4123, total length I 4.1, II 3.6, III 2.4, IV 4.3. Claw of leg with five teeth (Fig. 5F). Abdomen length 0.65. Colulus rectangular with five long bristles (Fig. 5C). Internal genitalia with long spermathecae narrowed at tip, conspicuous pore-plate at base; medial columnar uterus externus long, with visible internal chambers. Uterus externus shorter than spermathecae. Oval pore-plates on spermathecae with approximately 20–30 glandular ducts (Fig. 2C, D).

##### Variation.

Ten males: total length 1–1.25; carapace 0.4–0.5; femur I 1.05–1.4; ten females: total length 1–1.3; carapace 0.4–0.5; femur I 1–1.3.

##### Etymology.

The specific name Ritxòkò means “ceramic dolls” in the female language of the Karajá people, an indigenous population of the region. The dolls are produced by Karajá women, who model, burn, paint, and sell them (Silva 2015).

##### Natural history.

*Ochyrocera ritxoco* sp. nov. is a small troglobitic spider that is exclusive to caves in the Carajás karst region. Specimens were found only in aphotic zones of caves. They build small, tangled webs under blocks of stone on the ground or in slits (Fig. 6). The observed sex ratio for the species was 2.4F:1M (*N* = 66). *Ochyrocera ritxoco* sp. nov. was generally found in large cavities with horizontal projections varying from 9.5 to 216 meters (*N* = 13, mean = 107 m). All caves where the species was found have only one entrance and are located only in the middle and high slopes of Serra Norte. Most caves have aphotic zones or twilight zones (except for cavities N1_0103 and N1_0084) and high humidity, thus explaining the observed presence of small bodies of water in almost all cavities, especially during the wet season. The number of troglobitic species in these caves varied from one to ten (average 5.3 per cave), with species of the following taxa: spiders – Oonopidae (many species), Caponiidae (*Carajas paraua* Brescovit & Sánchez-Ruiz, 2016) and Ochyroceratidae (*Speocera* spp.); pseudoscorpions – Bochicidae, Chthoniidae and Ideoroncidae; springtails – Paronellidae (*Trogolaphysa* sp., *Cyphoderus* sp.), Entomobryidae (*Pseudosinella* sp.) and Sminthuridae (*Pararrhopalites* sp.); beetles – Carabidae (*Coarazuphium* spp.), Dytiscidae (*Copelatus cessaima* Caetano, Bena & Vanin, 2013); isopods – Scleropactidae (*Circoniscus* spp.), Calabozoidae; amphipods – Bogidiellidae (*Bogidiella* sp.); planarian – Prorhynchidae (*Geocentrophora* sp.); and Harvestmen – Escadabiidae.

##### Distribution.

Known exclusively from caves in a range of approximately 15 km of the Serra Norte (North Mountain), FLONA de Carajás, Parauapebas, state of Pará, northern Brazil (Fig. 10).

#### Ochyrocera ritxoo sp. nov.

Taxon classificationAnimaliaAraneaeOchyroceratidae

968D35C3-CEA4-523D-A92B-B62CC63F4ED0

http://zoobank.org/F161E5C9-B893-46D7-B724-737B01AF5705

Figs 7 , 8 , 9 , 10

##### Type material.

***Holotype*** male from Cave S11C_0201 (6°22'01"S, 50°23'07"W), FLONA de Carajás, Canaã dos Carajás, Pará, Brazil, 27/VII/2015, BioEspeleo Consultoria Ambiental col. (IBSP 193194). ***Paratype*** female from Cave S11C_0052 (6°23'56"S, 50°22'46"W), FLONA de Carajás, Canaã dos Carajás, Pará, Brazil, 09/III/2016, BioEspeleo Consultoria Ambiental col. (IBSP 193196),

##### Other material examined.

Brazil. Pará: Canaã dos Carajás, FLONA de Carajás, Cave S11C_0194 (6°24'20"S, 50°23'34"W), 12/III/2016, 1♂ (IBSP 193078); Cave S11C_0046 (6°24'02"S, 50°22'43"W), 19/IV/2016, 1♀ imm. (IBSP 193083), all collected by BioEspeleo Consultoria Ambiental; Cave S11D_0064 (710) (6°23'31"S, 50°18'48"W), 13–30/I/2010, R. Andrade & I. Cizauskas et al. col., 1♂ 1♀ 3 imm. (IBSP 174071); 10–19/V/2011, D. Bebiano col., 1♀ (IBSP 196512); Cave S11D_0064 (710) (6°23'31"S, 50°18'48"W), 13–30/I/2010, 1♀ (IBSP 196513); 01–14/VII/2010, R. Andrade & I. Cizauskas et al. col., 2♀ (IBSP 196514); 2♂ (IBSP 196515; SEM); Cave S11D_0096 (742) (6°23'37"S, 50°19'27"W), 13–30/I/2010, 1♂ (IBSP 196481); Cave S11D_0064 (710) (6°23'31"S, 50°18'48"W), 13–30/I/2010, 1♀ (IBSP 196482); Cave S11D_0064 (710) (6°23'31"S, 50°18'48"W), 1–14/VII/2010, 1♂ 1♀ (IBSP 196483) all collected by R. Andrade & I. Cizauskas et al.; Cave S11B_078 (6°21'33"S, 50°23'54"W), 28/IX/2018, Ativo Ambiental col. 1♂ (IBSP 260308).

##### Diagnosis.

*Ochyrocera ritxoo* sp. nov. is distinguished from *O. ritxoco* sp. nov. by having the embolus as long as the bulb of the male palp (Figs 7C–F, 8A, B) while *O. ritxoco* sp. nov. have an elongated embolus, which is two times longer than the bulb in the male palp (Fig. 1F), and by a short and striped distal area of the spermathecae and a shorter columnar uterus externus with approximately 3–4 internal chambers in the female genitalia (Fig. 8C, D) while the other species have a long and globose distal area in the spermathecae and an elongated columnar uterus externus with approximately eight internal chambers (Fig. 2C, D).

##### Description.

**Figure 9.**
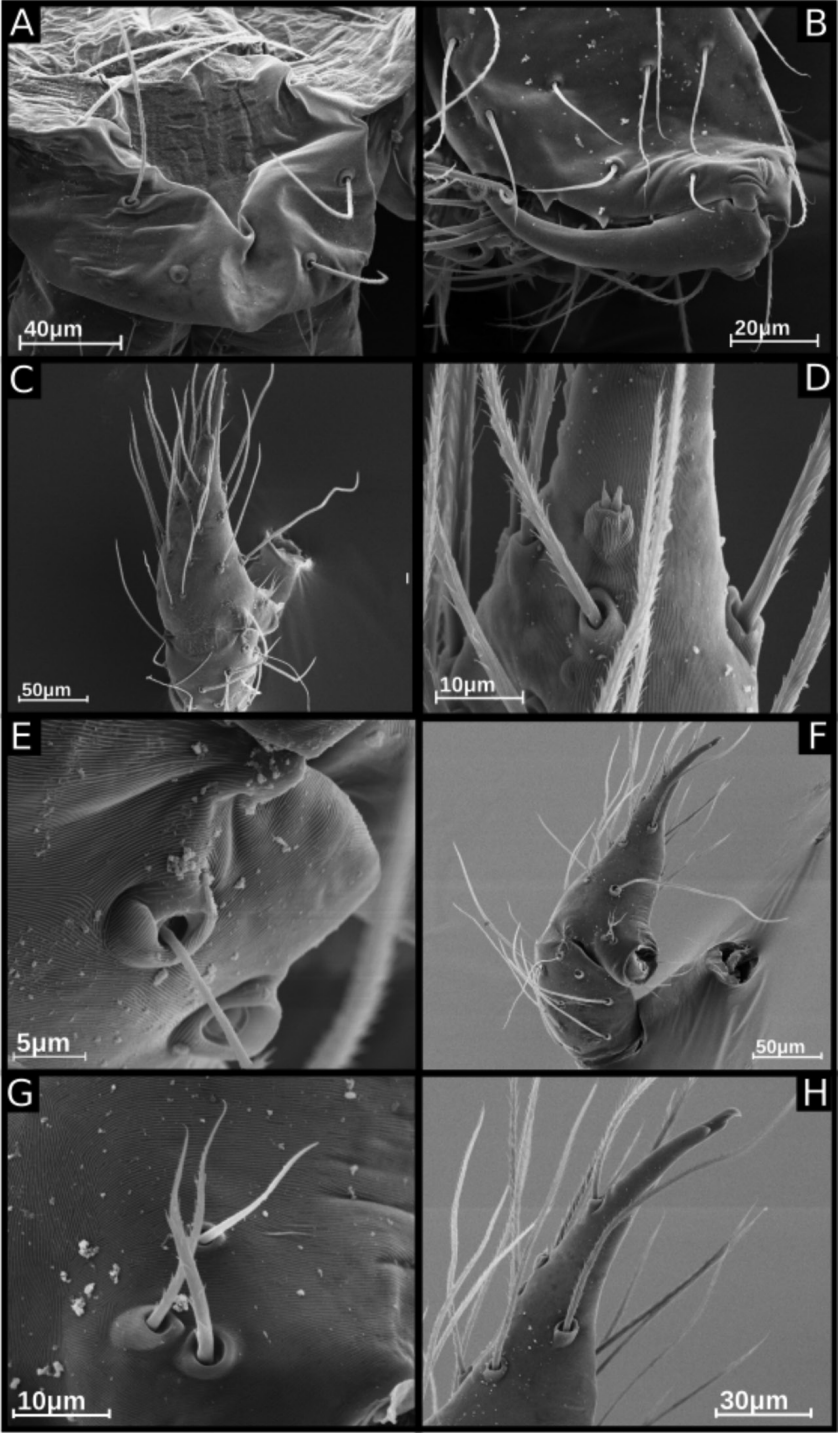
SEM images of *Ochyrocera ritxoo* sp. nov., male IBSP 196515 (**A–H**) **A** carapace, dorsal view **B** chelicerae, frontal view **C** cymbium, dorsal view **D** same, tarsal organ, detail **E** tibia of palp, trichobothria, apical dorsal view **F** cymbium, prolateral view **G** same, basal macrosetae, retrolateral view **H** same, cuspule, prolateral view.

**Male** (IBSP 193194) Total length 0.90. Carapace length 0.40, ovoid, narrowing gradually anteriorly, cream-colored and bright, pars cephalic flat, fovea absent (Fig. 7A). Clypeus with two pairs of long bristles (Fig. 9A). Eyes absent. Chelicerae light yellow, promargin with three teeth attached to a very long lamina (Fig. 9B); retromargin without teeth. Sternum cream-colored. Endites and labium as for *O. ritxoco* sp. nov.. Legs cream-colored, formula 1423, total length I 5.6, II 5.1, III 4.2, IV 5.4. Male palp with palpal femur length 0.03, palpal tibia enlarged, shorter than cymbium, with two long dorsal trichobothria (Fig. 8E), cymbium enlarged basally, slightly curved distally, bearing short apical cuspule; paired long setae on non-projected bases retrolaterally, elongated tarsal organ as in *O. ritxoco* sp. nov., basal setae on the rounded cymbial prolateral extension (Fig. 9F–H), bulb oval, embolus flattened, as long as cymbium (Fig. 7C–F, 8A, B). Abdomen length 0.6, oval, uniformly gray. Six epiandrous spigots with a short base.

**Female.** (Paratype IBSP 193196). Total length 1.0. Carapace length 0.35, as in male (Fig. 7B). Pedipalp without claw, with conical tip and subdistal trichobothrium. Clypeus, eyes, chelicerae, sternum, endites, and labium as in male. Legs as in male, formula 4123, total length I 5.3, II 4.9, III 4, IV 5.4. Abdomen length 0.65. Colulus triangular with six long bristles. Internal genitalia with enlarged spermathecae under the small pore-plate; medial columnar uterus externus short, internally with few visible chambers. Uterus externus ending in a narrow neck. Oval pore-plates on spermathecae with approximately 10–20 glandular ducts (Fig. 8C, D).

##### Etymology.

The specific name Ritxòò also means “ceramic dolls” but in the male language of the Karajá people, an indigenous population of the region. The making of these dolls, however, is an exclusive activity of women (Silva 2015).

##### Natural history.

*Ochyrocera ritxoo* sp. nov. is a small troglobitic spider that is exclusive to caves in the Carajás karst region. Specimens were collected only in aphotic zones of caves. The observed sex ratio for the species was 1.4F:1M (*N* = 17). *Ochyrocera ritxoo* sp. nov. was generally found in large cavities with horizontal projections varying from 26 to 245 meters (*N* = 7, mean = 102 m). These caves have one to three entrances and are located in all compartments of the Serra Sul landscape (top, high, medium and low slopes). All caves have aphotic zones and other troglobitic species were found in most caves, with the richness of troglobitic species per cave varying between one and six (average of four). The following taxa were found: spiders – Gnaphosidae (*Paracymbiomma carajas* Rodrigues, Cizauskas & Rheims, 2018), Caponiidae (*Carajas paraua* Brescovit & Sánchez-Ruiz, 2016) and Tetrablemmidae (*Matta* sp.); tailless whip scorpions – Charinidae (*Charinus ferreus* Giupponi & Miranda, 2016); pseudoscorpions, Chthoniidae; diplopods – Glomeridesmidae (*Glomeridesmus* sp.), Pyrgodesmidae, Pseudonannolenidae (*Pseudonannolene* spp.); springtails – Sminthuridae, Paronellidae; and beetles – Staphylinidae (Pselaphinae).

##### Distribution.

**Figure 10.**
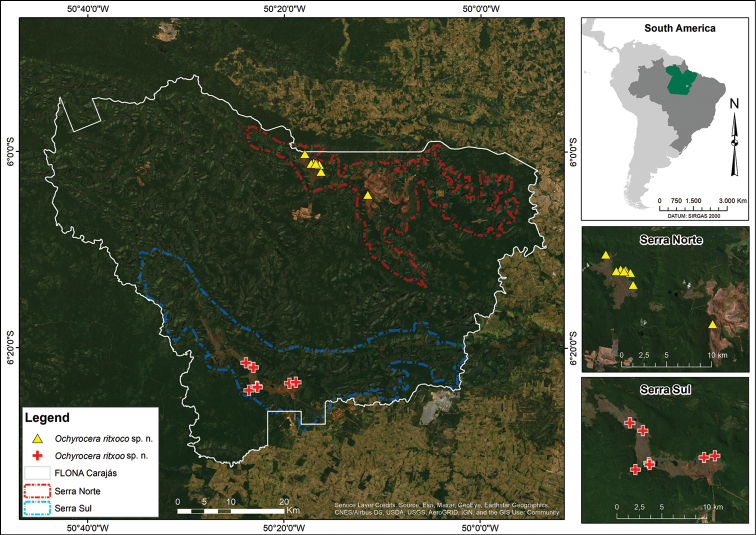
Distribution map of *Ochyrocera ritxoco* sp. nov., yellow triangles, and *Ochyrocera ritxoo* sp. nov., red plus signs, in FLONA de Carajás, Pará, Brazil.

Known exclusively from caves in a range of approximately 10 km of the Serra Sul (South Mountain), FLONA de Carajás, Canaã dos Carajás, state of Pará, northern Brazil (Fig. 10).

## Discussion

The fauna of subterranean spiders of the family Ochyroceratidae located in caves in Brazil is represented mainly by specimens of the genera *Ochyrocera* Simon, 1892, *Speocera* Berland, 1914, and *Theotima* Simon, 1893. The first two genera have troglomorphic spiders among their representatives and are a significant part of the yet unknown diversity of subterranean spiders in these environments (Baptista 2003). The genus *Ochyrocera* stands out for its abundance and diversity of species in ferruginous ecosystems, such as the Carajás system of the present study, with species colonizing the edaphic zone to the deep cave environment (Brescovit et al. 2018).

Both *Ochyrocera ritxoco* sp. nov. and *O. ritxoo* sp. nov. are troglobitic spiders that are restricted to iron formation caves in FLONA de Carajás. The description of these spiders expands the number of the endemic subterranean species (troglobites) and, together with *Carajas paraua* Brescovit & Sánchez-Ruiz, 2016 (Caponiidae) and *Paracymbiomma caecus* and *P. bocaina* (Gnaphosidae; Rodrigues et al. 2018), is evidence of a diversified subterranean araneofauna in the Carajás ferruginous system which must be considered in projects aimed at conservation and sustainable use of its natural resources.

## Supplementary Material

XML Treatment for Ochyrocera ritxoco

XML Treatment for Ochyrocera ritxoo
